# Review of 14 drowning publications based on the Utstein style for drowning

**DOI:** 10.1186/s13049-018-0488-z

**Published:** 2018-03-22

**Authors:** Allart M. Venema, Anthony R. Absalom, Ahamed H. Idris, Joost J. L. M. Bierens

**Affiliations:** 1 Department of Anaesthesiology, University Medical Center Groningen, University of Groningen, Hanzeplein 1, Huispostcode EB 32, Postbus 30001, 9700 RB Groningen, The Netherlands; 2 University of Texas Southwestern Medical Center, 5323 Harry Hines Blvd, Dallas, TX 75390-8579 USA; 3 Research Group Emergency and Disaster Medicine, Vrije Universiteit Brussels, Faculty of Medicine & Pharmacy, Laarbeeklaan 103, 1090 Brussels, Belgium; 4 Koninklijke Maatschappij tot Redding van Drenkelingen, Rokin 114, 1012 LB Amsterdam, The Netherlands

**Keywords:** Drowning, Utstein, Resuscitation

## Abstract

**Background:**

The Utstein style for drowning (USFD) was published in 2003 with the aim of improving drowning research. To support a revision of the USFD, the current study aimed to generate an inventory of the use of the USFD parameters and compare the findings of the publications that have used the USFD.

**Methods:**

A search in Pubmed, Embase, the Cochrane Library, Web of Science and Scopus was performed to identify studies that used the USFD and were published between 01-10-2003 and 22-03-2015. We also searched in Pubmed, Embase, the Cochrane Library, Web of Science, and Scopus for all publications that cited the two publications containing the original ILCOR advisory statement introducing and recommending the USFD. In total we identified 14 publications by groups that explicitly used elements of the USFD for collecting and reporting their data.

**Results:**

Of the 22 core and 19 supplemental USFD parameters, 6–19 core (27–86%) and 1–12 (5–63%) supplemental parameters were used; two parameters (5%) have not been used in any publication. Associations with outcome were reported for nine core (41%) and five supplemental (26%) USFD parameters. The USFD publications also identified non-USFD parameters related to outcome: initial cardiac rhythm, time points and intervals during resuscitation, intubation at the drowning scene, first hospital core temperature, serum glucose and potassium, the use of inotropic/vasoactive agents and the Paediatric Index of Mortality 2-score.

**Conclusions:**

Fourteen USFD based drowning publications have been identified. These publications provide valuable information about the process and quality of drowning resuscitation and confirm that the USFD is helpful for a structured comparison of the outcome of drowning resuscitation.

## Background

In 2003, a consensus-based ILCOR advisory statement recommended the Utstein style for drowning (USFD) for use in planning of, and reporting of the results of drowning studies to improve the quality and comparability of drowning studies [1, 2]. Since 2003 the USFD has been used to study drowning, which is a leading cause of accidental death worldwide, with an estimated death toll of 372.000 persons per year [3–17]. The USFD template consists of 22 core parameters that the statement recommended should be included in all drowning studies, and 19 supplemental parameters that are considered to be less important or are difficult to collect (Table 1).

**Table 1:** Overview of core and supplemental USFD parameters

	USFD parameters	Core/*Supplemental*
Victim Information	Victim identifier	Core
	Gender	Core
	Age	Core
	*Race or Ethnic category*	*Supplemental*
	Date and time of day of incident	Core
	*Residence*	*Supplemental*
	Precipitating event	Core
	*Preexisting illness*	*Supplemental*
Scene information	Witnessed	Core
	Body of water	Core
	*Water/liquid type*	*Supplemental*
	*Approximate water temperature*	*Supplemental*
	*Time of submersion*	*Supplemental*
	*Time of removal of victim from water*	*Supplemental*
	Unconscious when removed from water	Core
	*Cyanosis*	*Supplemental*
	Resuscitation before EMS arrived	Core
	*Method of CPR*	*Supplemental*
	EMS called	Core
	*EMS vehicle dispatched*	*Supplemental*
	*Time of first EMS assessment*	*Supplemental*
	Initial vital signs	Core
	*Oxygen saturation, temperature, blood pressure, pupillary reaction*	*Supplemental*
	Time of first EMS resuscitation attempt	Core
	Neurological status	Core
Emergency Department Evaluation and Treatment	Vital signs	Core
	Oxygen hemoglobin saturation	Core
	Arterial blood gas analysis, if unconscious or SaO2 < 95% on room air	Core
	Initial neurological status	Core
	*Pupillary reaction*	*Supplemental*
	Airway and ventilation requirements	Core
	*Toxicology testing*	*Supplemental*
Hospital Course	Airway and ventilation requirements	Core
	*Serial neurological function (admission, 6 h, 24 h, 72 h, discharge)*	*Supplemental*
	*Complicating illnesses*	*Supplemental*
Disposition	Alive or dead	Core
	Date of hospital discharge	Core
	Neurological outcome at hospital discharge	Core
	*Quality of life*	*Supplemental*
	*Cause of death*	*Supplemental*
	*Other injuries and morbidities*	*Supplemental*

The aim of this study was to generate an inventory of USFD usage during the more than 10 years since it was published, and to review and compare the findings of the publications that have used the USFD. This study elaborates on an initial study that was performed to support the revision process that generated the revised USFD, published in 2017 [18, 19]. Both the revised USFD and this current study will contribute to improvements in the uniformity of data collection and reporting, as recommended by the WHO in their recent document “Global Report on Drowning: preventing a leading killer” [17].

## Methods

A literature search was performed to identify peer-reviewed publications, concerning drowning that had used the USFD [1, 2] and were published between 01-10-2003 (month of the publication of the USFD) and 22-03-2015 (the latter date was arbitrarily chosen and was shortly before the revision process of the USFD was completed).

Pubmed search terms were: "Drowning"[Mesh] OR drown*[tw]) AND utstein[tw]; Embase search terms were: 'drowning'/exp. OR drown*:ab,ti AND utstein:ab,ti; Cochrane Library and Web of Science search terms were: drown* AND utstein; the Scopus search term used was “drowning AND Utstein”.

**Fig. 1:**
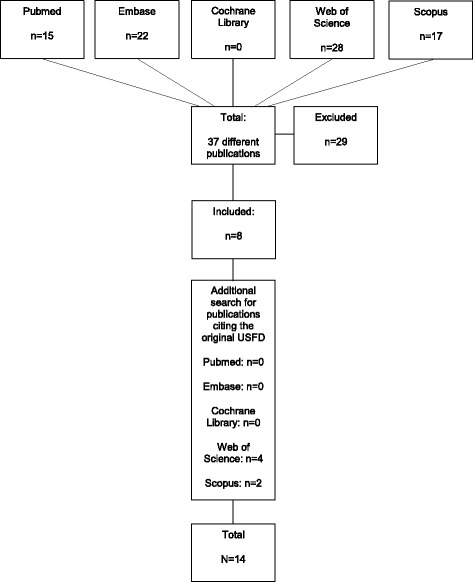
Flowchart of search strategy

The search provided 15 publications from Pubmed, 22 from Embase, none from the Cochrane Library, 28 from Web of Science and 17 from Scopus. Altogether, the systematic literature search identified 37 different publications (Fig. 1). These publications were independently reviewed by two authors (AV and AA), to identify those that explicitly stated an a priori intention to base their study on the USFD. After exclusion of drowning publications that did not explicitly mention that data collection was based on the USFD parameters, eight USFD based drowning publications remained [3–10]. Of the 29 publications that had not used the USFD, three were drowning publications of which data collection was based on the Utstein style for out of hospital cardiac arrest [20, 21], or did not describe the use of any Utstein template [22], four were publications on resuscitation that included only a few drowning patients [23–26], one was a resuscitation publication that excluded drowning victims [27], five were reviews [28–32], and 16 were other types of publications (definition of drowning, editorials, abstracts, letter to the editor, non-English language articles, post mortem examinations, book chapter) [33–48].

To enhance the robustness of the search, we additionally searched in Pubmed, Embase, the Cochrane Library, the Web of Science and Scopus for all publications that cited the two publications containing the original ILCOR advisory statement introducing and recommending the USFD [1, 2]. This revealed a total of six further publications that matched the inclusion criteria for our study, but had not been identified by the initial search strategy [11–16].

A total of 14 publications were thus included in the current analysis [3–16]. The individual publications were subsequently analysed independently by two reviewers (AV, AA) to identify the USFD parameters used by each publication, the information these USFD parameters provided, and USFD as well as non-USFD parameters related to outcome. In case of disagreement, discussion continued until agreement was reached.

We decided a priori to report all parameters for which an association with outcome was published, regardless of the number of publications in which they were used and to limit the reporting of non-outcome related parameters to those parameters that had been reported in five or more publications. Because the objective of our study was to report the use of the USFD, it was also decided a priori not to combine the data of the publications for a meta-analysis or to perform any statistical analysis of the patient data [49].

## Results

Table 2 summarises the 14 USFD drowning publications, in which 27–86% of the core and 5–63% of the supplemental USFD parameters were used.

**Table 2:** Fourteen drowning publications and their use of the 22 core and 19 supplemental USFD parameters

Study	Year of publication	Country	Study description	Victims	Category	USFD parameters	
				(*N*)	Adults/Children	Core (%)	Supplemental (%)
Eich et al. [3]	2007	Germany	Retrospective chart review on out of hospital cardiac arrest (OHCA) due to drowning and sustained resuscitation with CPB	12	Children	19 (86.4%)	11 (57.9%)
Grmec et al. [4]	2009	Slovenia	Retrospective chart review comparing the data of 528 primary cardiac arrest victims with 32 drowning victims	32	Adults	8 (36.4%)	3 (15.8%)
Youn et al. [5]	2009	South Korea	Prospective study on OHCA due to drowning	131	Adults and Children	19 (86.4%)	8 (42.1%)
Venema et al. [6]	2010	Netherlands	Retrospective study on bystander rescue and resuscitation	343	Adults and Children	14 (63.6%)	12 (63.2%)
Choi et al. [7]	2012	South Korea	Retrospective chart review on OHCA due to drowning and treatment with therapeutic hypothermia	20	Adults	15 (68.2%)	8 (42.1%)
Vähätalo et al. [8]	2014	Finland	Retrospective chart review of drowning children under 16 years of age who were hospitalized or died due to drowning	58	Children	12 (54.5%)	4 (21.1%)
Joanknegt et al. [9]	2015	South Africa	Retrospective chart review of drowning victims to inform prevention strategies	75	Children	13 (59.1%)	5 (26.3%)
Kieboom et al. [10]	2015	Netherlands	Retrospective chart review on hypothermic drowning victims with OHCA	160	Children	13 (59.1%)	5 (26.3%)
Hunsucker et al. [11]	2011	USA	Retrospective chart review on rescue reports of drowning victims with loss of spontaneous respiration in a waterpark environment	32	Adults and Children	6 (27.3%)	2 (10.5%)
Allan et al. [12]	2010	USA	retrospective chart review on US military drowning victims in in Iraq	8	Adults	10 (45.5%)	11 (57.9%)
Ma et al. [13]	2010	China	matched case control study on non-fatal drowning	325	Children	7 (31.8%)	1 (5.3%)
Wanscher et al. [14]	2012	Denmark	Retrospective review of a boating accident involving drowning victims and non-drowning victims with accidental hypothermia.	15	Adults and Children	18 (81.1%)	10 (52.6%)
Orlowski et al. [15]	2012	USA	Retrospective chart review on drowning occurring during a visit to relatives or friends	100	Children	8 (36.4%)	3 (15.8%)
Champigneulle et al. [16]	2015	France	Retrospective chart review on drowning victims with OHCA treated with extracorporeal life support	43	Adults and Children	17 (77.3%)	9 (47.4%)

Table 3 describes the nine core (41%) and five supplemental (26%) USFD parameters related to outcome. Table 4 describes the 11 core (50%) and six supplemental (32%) USFD parameters not related to outcome that were reported in 5 or more of the publications. In total, 10 USFD parameters not related to outcome were reported in less than 5 of the 14 drowning publications: Race or Ethnic category (*n* = 2), Residence (*n* = 4), Time of removal of victim from water (*n* = 3), Cyanosis (*n* = 4), Time of first emergency medical services (EMS) assessment (*n* = 4), Oxygen haemoglobin saturation (*n* = 3), Toxicology testing (*n* = 0), Date of hospital discharge (*n* = 1), Quality of life (*n* = 0), Other injuries and morbidities (*n* = 3).

**Table 3:** Overview of the USFD parameters related to outcome

USFD parameters *(Core/Supplemental)*	Total use (*n*)	Overall description	Significant relation with outcome
Victim Information			
Age *(Core)*	14	Age is described as mean, median or range: Mean age 3 years and 5 months-47.5 years [3–8, 12, 16]; median age 2–2.2 years [9, 10]; Range 1–60 years [11, 13–15].	One publication shows that survivors are significantly younger than non-survivors (38.9 ± 12.6 versus 56.6 ± 18.7; *p* = 0.03) [4].
Date and time of day of incident *(Core)*	6/9	Six publications describe the time of day [3, 5, 6, 8, 13, 14] and nine publications describe the season the drowning occurred in [3, 5–8, 10, 11, 13, 14]. Four publications describe that 39–100% of drownings occur during the daytime [3, 5, 8, 13] One publication reports that 67% of drownings occur between 12 AM and 8 PM and 33% of drownings from 9 PM to 11 AM [6]. Forty-seven to One hundred percent of drowings are reported to occur between springtime and the end of the summer [3, 5–8, 10, 11, 13].	One publication shows that outcome after drowning is significantly better in winter compared to other seasons: odds ratio 4.6 (1.4–15.1), *p* = 0.013 [10].
Scene information			
Witnessed *(Core)*	10	The drowning event is not witnessed in the majority of cases (58–81%) in four publications [3, 4, 8, 9]. In another five publications the drowning is witnessed in the majority [5–7, 10, 16]. In one publication the drowning was witnessed in 100% (by fellow victims) [14].	One publication shows that survivors are more likely to have had a witnessed drowning event than non-survivors (76 versus 61%; *p* = 0.036) [5].
Approximate water temperature *(Supplemental)*	6	In one publication a median water temperature for survivors of 20.4 (Interquartile range 13.7, 27.0) and 20.0^0^ C (interquartile range 8.6, 23.8) was reported (*p* = 0.184) [8]. In three publications a large range (0–28^0^ C) was reported [3, 10, 16]. In one study the water temperature was 2^0^ C for all victims [14]. In one publication the water temperature was described as warm (14%), cold (84%, or ice-cold (3%) [6].	In one publication the water temperature was lower for 24 h survivors among victims treated with ECLS (*p* = 0.04) [16].
Time of submersion *(Supplemental)*	10	The duration of submersion is described in different ways (median, mean, ordinal) but ranges from < 1 to 45 min [3, 5–12]. One publication described a submersion time of 10 min or more in 2% of the victims [15].	A longer duration of submersion is significantly associated with bad outcome in four publications [5, 8–10]. Two publications report no significant difference [3, 7].
Resuscitation before EMS arrived *(Core)*	10	Resuscitation attempts before the arrival of EMS varies between 24 and 93% [3–6, 8–11]. In one publication this parameter was described in 19% of the cases, of which 79% were resuscitated before EMS arrival [15]. In one publication BLS was immediately started after removal from the water in 100% of the victims by police officers or firefighters before ALS providers arrived [16].	One publication shows that drowning victims that survive have significantly more bystander CPR than non-survivors (57 versus 17%; *p* = 0.03) [4]. A significant relation between bystander resuscitation and outcome is not found in three publications [5, 8, 10].
Oxygen saturation, temperature, blood pressure, pupillary reaction *(Supplemental)*	6	In one publication at least one of these four parameters information is available in 4 out of 343 victims [6]. Hypothermia is reported in four publications [3, 12, 14, 16]. Pupillary reactions are described in three publications [3, 9, 12].	In one publication unresponsive and dilated pupils is significantly related with bad outcome (*p* < 0.001) [9]. In one publication the first prehospital core temperature was lower in the 24 h survivors among patients treated with ECLS (*p* = 0.07) [16]. In this same publication the association between a first prehospital core temperature of ≤26^0^ C and serum potassium level between 4.2 and 6.0 identified 24 h survivors among patients treated with ECLS with 100% sensitivity (95% CI: 28–100%) and specificity (95% CI: 71–100%).
Time of first EMS resuscitation attempt *(Core)*	3	The mean time interval is described in two publications [5, 7]. In another publication this parameter is included, but the information is not available in any victims [6].	The time of first EMS resuscitation is found to be significantly associated with outcome in one publication: 11.2 ± 5.6 min in survivors versus 21.4 ± 12.8 min in non-survivors; *p* = < 0.001 [5].
Emergency Department Evaluation and Treatment			
Vital signs *(Core)*	8	Vital signs are absent in 20 to 100% of the victims on arrival at the ED [3–5, 7, 9, 10, 14]. In one publication all victims had asystole at arrival at the ED [16].	In one publication resuscitation at arrival in the ED is negatively associated with outcome (*p* < 0.001, OR 0.03, 95% CI 0.01–0.13) [9]. In the same publication hypothermia is significantly related to bad outcome (*p* < 0.001, OR 18.00, 95% CI 3.35–96.74). In one publication 24 h survivors among patients treated with ECLS had a significantly lower in hospital initial core temperature (*p* = 0.004) [16].
Arterial blood gas analysis, if unconscious or SaO2 < 95% on room air *(Core)*	8	Hypoxemia, acidosis, and hypercarbia are common findings [3, 5, 7–10, 14, 16].	The more severe the acidosis, the worse outcome is (*p* < 0.001–0.014) [8–10]. In one publication an initially lower pH relates to hospital mortality (*p* = 0.008) [7]. Drowning cardiac arrest victims have a higher initial pCO2 compared to non-drowning victims in cardiac arrest (*p* < 0.001). Endtidal CO2 after 1 min of CPR (p 0.02) and the final endtidal CO2 (*p* < 0.001) were independent factors for survival [10]. Less negative base excess is related to better outcome. (*p* < 0.001–0.001) [8, 10].
Initial neurological status *(Core)*	8	The GCS is 3 at the ED in all patients in three publications (in only one patient in one of these studies a palpable pulse was reported) [3, 5, 7]. In one publication the median Glasgow Coma Score (GCS) ranged between 11.5 in survivors and 3 in non-survivors [8]. In one publication the GCS was described as < 5 (41% good outcome, 18% neurologic sequelae, 41% death) or ≥ 5 (98% good outcome, 2% death) [9]. In one publication the median GCS was 3 [10]. In one publication the GCS ranged from 3 to 15 [14]. One study described that none of the victims had clinical signs of life [16].	A low GCS is significantly associated with bad outcome in three publications (*p* < 0.001) [8–10].
Pupillary reaction *(Supplemental)*	4	In three publications, fixed and dilated pupils were reported in 47%, 95% and 100% of victims [5, 7, 14]. In one publication pupillary reactions are described as reactive (*n* = 44), sluggish (*n* = 6), unreactive not dilated (*n* = 6), or unreactive dilated (*n* = 5) [9].	Unreactive dilated pupils in the ED are significantly related to bad outcome in one publication (*p* < 0.001; OR 0.01; 95% CI 0.04–0.23) [9].
Airway and ventilation requirements *(Core)*	7	In six publications 100% of the patients are ventilated mechanically or manually [3, 5, 7, 9, 10, 16]. In one publication it was reported that one patient was intubated and ventilated [14].	Intubation at the ED (*p* = 0.002) is significantly related to bad outcome in one publication [9].
Hospital Course			
Serial neurological function (admission, 6 h, 24 h, 72 h, discharge) *(Supplemental)*	3	In one publication myoclonic or seizure activity (including treatment with medications), loss of pupillary response, absent motor response to pain, somatosensory evoked potentials (SSEPs), and the use of brain imaging are described [7]. One publication only describes the use of a CT scan [12]. One publication describes the use of electroencephalographic recordings, SSEPs, magnetic resonance imaging and the use of biomarkers (neuron specific enolase and protein S100B) [14].	Neurological function testing, somatosensory evoked potentials (SSEPs), brain imaging (computed tomography or diffusion-weighted imaging) and neurological examination of motor response to motor response to pain after 3 days, are significantly related to bad outcome in one publication [7].

**Table 4:** The USFD parameters used in 5 or more of the 14 USFD drowning publications

USFD parameters *(Core/Supplemental)*	Total use (*n*)	Overall conclusions
Victim Information		
Victim identifier *(Core)*	6	Not related to a conclusion
Gender *(Core)*	14	Most drowning victims are male (57–100%) in 12 publications [3–6, 8–13, 15, 16]. In one publication 35% of the victims are male [7]. In one publication the distribution is unclear due to a contradiction in the reporting of this parameter [14].
Precipitating event *(Core)*	10	The most frequently reported precipitating events are motor vehicle or boating accidents, and swimming [6, 8, 12–14].
Pre-existing illness *(Supplemental)*	5	Pre-existing illness is either not reported or not linked to outcome in any of the publications [3, 5, 6, 8, 12].
Scene information		
Body of water *(Core)*	13	Drowning occurs mostly (66–100%) in natural bodies of water such as rivers, lakes and canals in eight publications [3, 5–8, 10, 12–14, 16]. Two publications report that drowning occur predominantly (52 and 95%) in private or public pools [9, 15]. Another publication has only included drowning in swimming pools [11].
Water/liquid type *(Supplemental)*	11	Seven publications predominantly (84–100%) report fresh water drownings [3, 5–8, 10, 16]. In one publication drowning in roadside ditches, canals and retention ponds are reported as the predominant water sources, however salinity was not determined [12]. In one publication all drownings occurred in salt water [14]. In the two publications where most drownings occurred in pools, the salinity of these pools was not specified [9, 15].
Unconscious when removed from water *(Core)*	8	Four publications report that 83–100% of the victims were unconscious when removed from the water [3, 5, 9, 16]. In two publications a minority were unconscious [6, 13]. Two publications show an equal distribution [12, 14].
Method of CPR *(Supplemental)*	6	One publication only reported that the victims were resuscitated according to guidelines [16]. Five publications described in more detail how resuscitation was performed, based on which it seems that international guidelines were followed [4–6, 11, 14]. One of these publications reported that in 10% resuscitation was not performed according to guidelines (tapping on back, rubbing abdomen, pressing water out of lungs etc.) [6]. Another one of these publications described the use of abdominal trusts in and outside the water [11].
EMS called *(Core)*	11	Calling the EMS is directly or indirectly reported in 11 publications but as such provides no relevant information.
EMS vehicle dispatched *(Supplemental)*	10	Dispatch of the EMS is directly or indirectly reported in ten publications but as such provides no relevant information.
Initial vital signs *(Core)*	5	Two publications directly or indirectly reported that all victims were in cardiac arrest [3, 16]. One publication reported that all victims had a Glasgow Coma Scale of 3 [5]. One publication reported that 53% of the victims were in cardiac arrest [14]. One publication described that the information was available in 0.3% of the victims but made no further specifications [6].
Neurological status *(Core)*	5	Two publications reported that all victims had a Glasgow Coma Scale of 3 [3, 5]. One of these publications also reported fixed dilated pupils for all patients [3]. One publication reported that 16% of the victims were unconscious and 84% were conscious [6]. One publication reported that four patients (50%) were comatose of which two had fixed dilated pupils with diffuse flaccid paralysis. This same publication also reported a mean initial Glasgow Coma Scale of 10.4 [12]. In one publication this information was indirectly available because all were in cardiac arrest [16].
Hospital Course		
Airway and ventilation requirements *(Core)*	7	All seven publications that reported this parameter only globally described it, which provided no real relevant information [3, 7, 8, 12, 14–16].
Complicating illnesses *(Supplemental)*	6	Six publications report complicating illnesses such as pneumonia, acute respiratory distress syndrome, pancreatitis, rhabdomyolysis, disseminated intravascular coagulation acute renal failure, multiple organ failure and septic shock [3, 5, 7, 9, 12, 16]. In one of these publications extubation stridor, minor neurologic deficits and corneal ulceration are reported which all are resolved at the time of discharge [9].
Disposition		
Alive or dead *(Core)*	14	Survival is good (16–93%) in most studies [3–12, 14, 15]. In one study only 5% of the victims survived [16]. One publication only reports non-fatal drowning [13].
Neurological outcome at hospital discharge *(Core)*	10	In four publications 7–20% had a (Paediatric) Cerebral Performance Scale/Category (P)CPC score of one to two [3, 5, 7, 8]. In one publication 85% of the patients had a CPC score of one to two [4]. In one publication 84% of the patients do not have any neurological complications, while 5.3% have irreversible neurological sequellae [9]. In one publication 11% of the patients have a (P)CPC score of three or less 1 year after the drowning incident [10]. In one publication hypoxic encephalopathy is reported in one victim (13%) [12]. One publication described a median Functional Independence Measure of 115 (range 51–121) and a median extended Glasgow Coma Scale score of 4 (range 3–7) after 6 months [14]. In one publication two victims (5%) survived to hospital discharge of which one had a CPC score (at discharge and at 6 months later) of one and one had a CPC score of three [16].
Cause of death *(Supplemental)*	5	Five publications described causes of death such as multi organ failure, septic shock, cardiac arrest, respiratoy failure, severe brain injury and brain death [3, 7, 10, 12, 16].

The USFD publications also included non-USFD parameters. The non-USFD parameters related to outcome are described below.

In one publication the first hospital core temperature of victims treated with extracorporeal life support (ECLS), 24 h survivors had a lower temperature than non-survivors (*p* = 0.004) [16].

Ten publications report the initial cardiac rhythm (at EMS arrival, at the emergency department (ED) and/or during hospital admission). With the exception of two studies [14, 16], the other studies showed that a shockable rhythm is rare (0–9%) in drowning victims in need of resuscitation [3–5, 7–10, 12]. Three of the latter publications showed a relation between the initial cardiac rhythm and outcome [3, 9, 10].

Eleven publications included parameters that were related to the start or duration of basic life support (BLS), advanced life support (ALS), or interventions by EMS, either as time intervals or as time points [3–5, 7–12, 14, 16]. An early start of resuscitation, rapid transfer to hospital and shorter duration of resuscitation were significantly related to better outcome [4, 5, 7, 10].

In one publication, intubation at the drowning scene was significantly related to bad outcome [9].

One publication concluded that glucose levels were significantly lower in survivors [8]. The relationship between serum potassium and outcome was inconsistent [3, 4, 8, 16].

The use of inotropes/vasoactive medications was related to outcome in three publications [4, 9, 10]. One of these publications concluded that more drowning victims that survived received vasopressin than non-survivors (64% versus 22%;*p* = 0.03) [4]. The use of inotropic/vasoactive agents was associated with worse outcome in two publications (*p* < 0.001 and *p* = 0.01) [9, 10].

The Paediatric Index of Mortality 2-score for patients admitted to the intensive care unit (ICU) has been calculated in one publication and was significantly higher in non-survivors [9].

In 4 of the 14 identified publications the authors commented on the USFD and/or suggested alterations to the USFD. One publication, involving a study in which identification of non-USFD outcome related parameters was a secondary study goal, recommended inclusion of information on early BLS, serum potassium, rewarming speed after the use of cardiopulmonary bypass and the initial cardiac rhythm in the USFD [3]. Another publication, involving a study in which the feasibility of the USFD parameters was tested as a secondary study goal, suggested changing some parameters on victim and scene information, including rescue related parameters and rescue related injuries [6]. In this same publication the authors also suggested use of ‘country of birth’ instead of the USFD parameter ‘race or ethnic category’ [6]. One publication suggested reporting ‘time of submersion’ as core parameter. Furthermore this same publication concluded that the USFD is applicable for reporting retrospective data of drowned children [8]. One publication promoted the USFD and suggested the creation of an international registry [16].

## Discussion

Between 1-10-2003 and 22-3-2015, the USFD has been used in 14 USFD based drowning publications, which involved different populations, focus and methodology. The cumulative findings of the reports have identified 14 USFD parameters for whom associations with outcome were reported. Also non-USFD parameters related to outcome have been identified: initial cardiac rhythm, time points and intervals during resuscitation, intubation at the drowning scene, first hospital core temperature, serum glucose and potassium, the use of inotropic/vasoactive agents and the Paediatric Index of Mortality 2-score.

None of the 14 USFD based drowning publications included in this review used all USFD parameters. This is explained in part by the narrow focus of some of the publications, for example on the pre-hospital setting [6], and the differences in methodology of the publications. Furthermore, the results show that several parameters such as the age of the drowning victims were described inconsistently (mean, median or range). These inconsistencies are probably the result of both the preferences in data reporting by the individual researchers, as well as the fact that the USFD does not provide extensive advice on this matter. Such inconsistencies hinder the goal of this template that is designed to improve the quality and comparability of individual studies, in order to identify ways in which outcome can be improved. Despite these issues, the use of the USFD parameters has enabled a comparison of resuscitation outcome data from these different drowning publications during the review process.

Several researchers have included in their research additional parameters potentially related to drowning outcome and some have recommended the inclusion of these additional parameters in drowning research.

This review of 14 USFD based studies was initiated at the start of the USFD revision process. Preliminary data were included in the consensus discussions that resulted in the revised USFD publications [18, 19]. The results we report here, support the decision that was made to revise the USFD [18, 19]. It is hoped that the revised USFD and this review will assist researchers in studying drowning and will encourage them to use the USFD parameters in their research. This might lead to a more complete use of the USFD and thereby improved comparability of drowning studies in the future. The results we report here will hopefully also help to inform future revisions to the USFD.

There are some limitations regarding the review process that should be mentioned. For the purpose of this review we have only focused on USFD based drowning publications. It is possible that other publications on drowning in the same time period might have demonstrated different results. By using the search terms and databases mentioned in the Methods section, the potential for missing relevant publications is minimized, but cannot be ruled out entirely.

The results of the reviewed publications displayed a variety of important information on the outcome and circumstances of drowning resuscitation. However, the authors realise that the USFD is only a tool to facilitate drowning resuscitation research. A recent publication concluded that the methodology of future drowning studies also needs to be based on unbiased high quality data and multi-variate analysis [49].

## Conclusions

Between 2003 and 2015, 14 USFD based publications on drowning have been published. These publications have identified associations between several USFD as well as non-USFD parameters and outcome. None of the publications reported all USFD parameters and there were inconsistencies in the way the USFD parameters were reported. This negatively influenced a structured comparison of the USFD parameters between the studies. However, despite the difficulties these publications still show that the use of the USFD is helpful for a structured comparison of the outcome of drowning resuscitation and provides valuable information about the process and quality of drowning resuscitation.

The analysis also points at some limitations of the USFD and has identified important additional parameters that have been considered for a revised USFD.

## Abbreviations

ALS: Advanced life support
BLS: Basic life support
ECLS: Extracorporeal life support
ED: Emergency department
EMS: Emergency medical services
ICU: Intensive care unit
USFD: Utstein style for drowning
WHO: World Health Organization
